# Draft Genome Sequence of Arthrobacter globiformis mrc11, an Antimicrobial Agent Isolated from a Khangkhui Cave Deposit

**DOI:** 10.1128/MRA.01620-18

**Published:** 2019-03-14

**Authors:** Dinabandhu Sahoo, N. Jusna Devi, N. Ngashangva, P. Momota, Y. Rojeena, S. Indira Devi

**Affiliations:** a Microbial Resources Division, Institute of Bioresources and Sustainable Development (IBSD), Department of Biotechnology, Government of India, Imphal, Manipur, India; Loyola University Chicago

## Abstract

Arthrobacter globiformis mrc11 was isolated from a Khangkhui cave deposit. Here, we report the draft genome sequence of this phylogenetically novel organism, which has a genome size of 4.89 Mb, a 65.9% GC content, and 4,657 predicted open reading frames that can be translated. This study will give insights into the mechanisms of the strain’s antimicrobial activity.

## Abstract (precis)

Arthrobacter globiformis mrc11 was isolated from a Khangkhui cave deposit. Here, we report the draft genome sequence of this phylogenetically novel organism, which has a genome size of 4.89 Mb, a 65.9% GC content, and 4,657 predicted open reading frames that can be translated.

## ANNOUNCEMENT

Antibiotic resistance of pathogenic bacteria has become one of the greatest threats to human health over the past few decades (1). Caves with surface entrances represent one of the unique and poorly studied ecosystems on Earth (2). The antimicrobial activity of microorganisms against pathogenic bacteria from different caves has been reported (3 – 5). Arthrobacter globiformis mrc11 was isolated from a Khangkhui cave deposit in the Ukhrul district of Manipur, India (25°03′11.9ʺN, 94°24′26.0ʺE), at 1,652 m above sea level. The cave deposit (soil) was collected, dissolved in sterile water, and serially diluted. The 200-μl diluents were inoculated on a Luria-Bertani agar plate and incubated at 30°C overnight. The visibly grown bacteria were preliminarily screened and selected for their antimicrobial properties by a spot-on lawn antimicrobial assay (6).

To our knowledge, this is the first strain reported from a cave deposit. The bacteria that showed antimicrobial activity on preliminary screening were grown overnight at 30°C, and the crude DNA was extracted using a HiPurA bacterial genomic DNA purification kit for the sequencing (7).

The genome sequencing was performed on the Illumina MiSeq/HiSeq (rapid) platform. Libraries were prepared with Illumina technology (Nextera XT DNA library preparation kit), and the adapter sequences were removed using Cutadapt version 1.8 (8). The genome had 21,637,904 raw reads (total reads) (forward and reverse strands), a size of 5,409.4 Mbp, a 31.5% GC content, and a 250-bp read length. All low-quality (Q < 30) data were filtered out using Sickle version 1.33 (9), and the clean reads were subjected to KmerGenie (10) to predict the optimal k value and assembly size. The cleaned reads consisted of 20,844,378 total reads, 4,226.03 Mbp (total number of bases), and a 34.33% GC content. *De novo* assembly was performed using ABySS version 2.0.1 with a k-mer range of 21 to 127 (11). Genes were predicted using the NCBI Prokaryotic Genome Annotation Pipeline version 4.5 (12). There were 4,512 total predicted genes; 4,442 coding genes; 70 RNAs, including 52 tRNAs, 15 rRNAs, and 3 noncoding RNAs (ncRNAs); and 157 pseudogenes. The draft genome assembly has 45.0× coverage and 105 scaffolds totaling 4,892,409 bp with an *N*_50_ value of 139,250 bp, an *L*_50_ of 10, and a GC content of 65.90% (13).

antiSMASH version 4.1.0 analysis of the mrc11 genome predicted 4 secondary metabolite gene clusters comprising siderophores, type III polyketide synthase, and two other siderophore gene clusters which have shown 80% similarity with desferrioxamine biosynthetic gene clusters and 19% similarity with streptomycin biosynthetic gene clusters (14). Rapid Annotation using Subsystem Technology tool kit (RASTtK) analysis of the genome predicted the important functional category of 18 membrane transport, 1 stress response, and 2 fatty acids; lipids; and isoprenoid. Since it is a new organism, no function for motility and chemotaxis has been assigned (15).

The functional comparison of the genome sequences revealed the closest neighbors of Arthrobacter globiformis mrc11 to be Arthrobacter chlorophenolicus A6 followed by Renibacterium salmoninarum ATCC 33209 and *Cryocola* sp. strain 340MFSha3.1 (15).

Thus, we concluded that Arthrobacter globiformis mrc11 harbors important putative secondary metabolites and other antibiotic genes, and further exploration will lead to significant contributions to antimicrobial drug development.

### Data availability.

This whole-genome shotgun project has been deposited in DDBJ/ENA/GenBank under the accession no. NZ_QLNP00000000. Raw sequencing reads have been deposited in the SRA under the accession no. SRR8529832.
